# Incidence rates of progressive childhood encephalopathy in Oslo, Norway: a population based study

**DOI:** 10.1186/1471-2431-7-25

**Published:** 2007-06-27

**Authors:** Petter Stromme, Oivind Juris Kanavin, Michael Abdelnoor, Berit Woldseth, Terje Rootwelt, Jorgen Diderichsen, Bjorn Bjurulf, Finn Sommer, Per Magnus

**Affiliations:** 1 Department of Pediatrics, Ullevål University Hospital and Faculty of Medicine, University of Oslo, Norway; 2 Centre for Clinical Research, Ullevål University Hospital and Faculty of Medicine, University of Oslo, Norway; 3 Department of Medical Biochemistry, Rikshospitalet-Radiumhospitalet Medical Center, Oslo, Norway; 4 Department of Pediatrics, Rikshospitalet-Radiumhospitalet Medical Center, Oslo, Norway; 5 Frambu Health Centre, Siggerud, Norway; 6 Norwgian Institute of Public Health, Oslo, Norway

## Abstract

**Background:**

Progressive encephalopathy (PE) in children is a heterogeneous group of diseases mainly composed of metabolic diseases, but it consists also of neurodegenerative disorders where neither metabolic nor other causes are found. We wanted to estimate the incidence rate and aetiology of PE, as well as the age of onset of the disease.

**Methods:**

We included PE cases born between 1985 and 2003, living in Oslo, and registered the number presenting annually between 1985 and 2004. Person-years at risk between 0 and 15 years were based on the number of live births during the observation period which was divided into four 5-year intervals. We calculated incidence rates according to age at onset which was classified as neonatal (0–4 weeks), infantile (1–12 months), late infantile (1–5 years), and juvenile (6–12 years).

**Results:**

We found 84 PE cases representing 28 diagnoses among 1,305,997 person years, giving an incidence rate of 6.43 per 100,000 person years. The age-specific incidence rates per 100,000 were: 79.89 (<1 year), 8.64 (1–2 years), 1.90 (2–5 years), and 0.65 (>5 years). 66% (55/84) of the cases were metabolic, 32% (27/54) were neurodegenerative, and 2% (2/84) had HIV encephalopathy. 71% (60/84) of the cases presented at < 1 year, 24% (20/84) were late infantile presentations, and 5% (4/84) were juvenile presentations. Neonatal onset was more common in the metabolic (46%) (25/55) compared to the neurodegenerative group (7%) (2/27). 20% (17/84) of all cases were classified as unspecified neurodegenerative disease.

**Conclusion:**

The overall incidence rate of PE was 6.43 per 100,000 person years. There was a strong reduction in incidence rates with increasing age. Two-thirds of the cases were metabolic, of which almost half presented in the neonatal period.

## Background

Progressive neurological disease in children poses an important challenge to our health system in terms of diagnosis and management [1-3]. In the present study, we focused on children presenting with signs of progressive CNS disease associated with impairment of cognitive functioning, designated progressive encephalopathy (PE). In the literature, PE is often used interchangeably with neurodegenerative encephalopathy. Both terms lack a firm definition, but we preferred PE because it encompasses clinically progressive conditions without demonstrable neuronal loss as well as those with a demonstrable loss of neural tissue, most often detected by magnetic resonance imaging (MRI) examination.

Although the different diseases causing PE are individually rare, their cumulative incidence has been estimated to be 0.5 per 1000 live births [4], which is within the same range as other major neurological impairments, such as neural tube defects [5] or infantile hydrocephalus [6], and approximately half the incidence of cerebral palsy in full term children [7]. PE is predominantly caused by inborn errors of metabolism, hereafter called metabolic diseases. PE also contains a relatively large mixed group of neurodegenerative disorders without an identifiable metabolic deficiency, hereafter designated neurodegenerative disease. Infectious, inflammatory or toxic aetiologies may also be considered as causes of PE. Epidemiological studies have most often focused on the cumulative incidence of known diagnoses associated with PE, particularly metabolic diseases. Cases with such diagnoses have either been discovered through screening programs for metabolic diseases or they have been registered after clinical presentation suggestive of metabolic deficiency [8-10]. The burden of disease may be heavily felt by patients and caregivers when the diagnosis remains unknown despite extensive investigation. It has been estimated that approximately one-third of all paediatric brain disorders without a known diagnosis, also called "anonymous" brain disorders, can be classified as PE [11]. As epidemiological data are scarce, we aimed to estimate the overall incidence rate of PE with its different aetiologies and to determine the age of onset of symptoms of the disease.

## Methods

### Case definition

In accordance with Uvebrant et al [12], PE was divided into metabolic, neurodegenerative, and infectious aetiology groups. The metabolic group was divided into disorders involved in metabolic processes related to subcellular organelles (lysosomes, mitochondrial respiratory chain, and peroxisomes) or defects of intermediate metabolism (organic acidurias, fatty acids oxidation disorders, urea cycle disorders, galactosemia, and unspecified intermediate metabolic defects). The latter group contained a few patients in whom specific management, such as a galactose free diet in galactosemia, protein restriction, and vitamin B12 supplementation in methylmalonic aciduria, and appropriate caloric supply in fatty acid oxidation defects, prevented them from developing clinical signs of disease progression.

The neurodegenerative group consisted of patients with progressive loss of neural tissue, where a metabolic or other aetiology could not be detected. This group was subdivided into a group of patients with a specified or known diagnosis and a group of patients in whom an aetiological diagnosis had not been identified despite extensive work up. The degenerative course of the disease in the unspecified degenerative group was documented with repeated clinical assessments and MRI examination of the brain showing atrophy of brain tissue. We classified patients according to the part of the CNS that was predominantly affected by the degenerative process: cerebral cortex, cerebral white matter, basal ganglia, cerebellum, and brain stem. The infectious group consisted of patients with encephalopathy caused by infectious agents, for example human immunodeficiency virus (HIV). We recorded the age of onset of symptoms of the disorder and the age when an aetiological diagnosis was established. In the patients with unspecified neurodegenerative disease, we assigned the time of "diagnosis" as the age when a down-hill course in each case was recognized. The age of onset was defined as neonatal (0–4 weeks), infantile (1–12 months), late infantile (1–5 years), juvenile (6–12 years), or late juvenile (>12 years).

We excluded patients with spinocerebellar ataxia and spinal muscular atrophy and other neuromuscular disorders if not accompanied by signs of encephalopathy. We did not include children with multiple sclerosis, as they usually do not exhibit disturbances of cognitive functioning in the early years. Developmental disorders such as Rett syndrome and autism were excluded. In Rett syndrome, there is a period of regression in the early phase. However, mental capacity does not continue to deteriorate in this disorder [13]. In autism, there may be a limited period of regression. However, autism spectrum disorders are not typically associated with progressive brain atrophy detected by MRI. The two diseases detected by the Norwegian newborn screening program, phenylketonuria and congenital hypothyroidism, were also excluded from the study, as affected individuals would not present with signs of encephalopathy.

### Search procedure

Before 1998, the population of Oslo was serviced by the paediatric departments at Aker and Ullevål University hospitals. Since their merger in 1998, Ullevål University Hospital has been the only paediatric ward servicing children between 0 and 15 years in Oslo. All medical diagnoses in children hospitalized at the two hospitals since 1985 have been stored in a common electronic registry of diagnoses (ERD). Because the national diagnostic laboratory for metabolic diseases is located at Rikshospitalet-Radiumhospitalet Medical Center (RRMC) in Oslo, we supplemented our ascertainment with the RRMC diagnostic data. All diagnoses in the ERD had been coded according to the International Classification of Diseases (ICD). When we conducted our search in the ERD, we retrieved the ICD code and birth year for each patient. We only considered children born between 1985 and 2003 living in Oslo during this period. The ERD was searched for codes in ICD-8 (before 1986) [14], ICD-9 (between 1986 and 1998) [15], and ICD-10 (after 1998) [16] compatible with PE (Table 1). The search resulted in a list of potential cases for the study. Subsequently, the medical charts and reports of the MRI examinations were critically reviewed (PS, OJK) to solely include patients who complied with our case definition criteria. We also consulted the Department of Pathology at Ullevål University Hospital to retrieve cases diagnosed at the post mortem examination with variant Creutzfelt-Jacobs disease (vCJD) or subacute sclerosing panencephalitis (SSPE).

**Table 1:** ICD codes used in the search for cases of progressive encephalopathy in children born between 1985 and 2003

Diseases	ICD-10 (>1998)	ICD-9 (1986–1998)	ICD-8 (<1986)
**Inborn errors of metabolism**			
Amino acidopathies	E70–E72	270	270
Carbohydrate metabolism	E74	271	271
Sphingolipids and other lipid storage disorders	E75	272	272
Glucoseaminoglycans (mucopolysaccharidoses)	E76	277.5	273
Glycoprotein metabolism	E77	277.5–277.9	270–273
Purin and pyrimidine	E79	277.2	270
Mineral metabolism	E83	275	273

**Other aetiological diagnoses**			
Systemic atrophies primarily affecting CNS	G10–G13	333	331
Extrapyramidal and other movement disorders	G20–G26	333	332
Other CNS degenerative diseases	G30–G32	340	340–341
Demyelinating disorders	G35–G37	340–341	340–341
HIV with dementia	B22	-	-

**Unspecified/Descriptive diagnoses**			
Unspecified metabolic disease	E88.9	NA	NA
Encephalopathy	G93.4; G93.9	348.3; 348.9	781.7
Other CNS disease	G96	348.3	NA

NA: Not available

### Incidence rates and cumulative incidences

**Figure 1:**
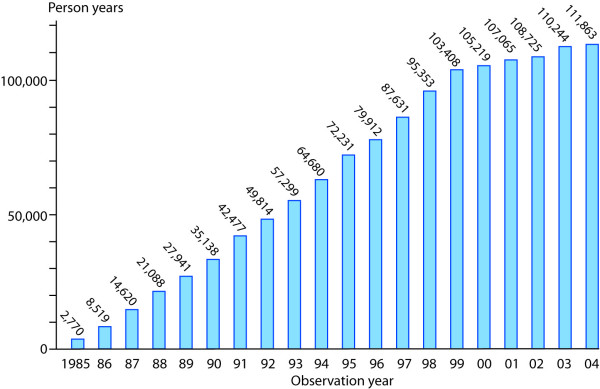
**Person years during the observation period**. The number of person years for each year increased gradually until 1999 when the at-risk population included the complete age cohort between 0 and 15 years.

As the admittance age to the paediatric departments had been limited to 15 years, we defined the age of the "at-risk" population for developing PE to be between 0 and 15 years of age. With a historical cohort design, only subjects born between 1985 and 2003 residing in Oslo were included. We registered new cases of PE having presented annually between 1985 and 2004, a 20-year observation period. In order to evaluate time trends, we divided the observation period into four periods: 1985–89, 1990–94, 1995–99, and 2000–04. Although the youngest patients were born in 2003, we chose to include 2004 as the last year of the observation period accepting the fact that ascertainment for this year could be an underestimate Incidence rates of PE were calculated as the number of cases divided by the number of person years at risk, expressed per 100,000 person years. The calculations of person years were based on the number of live births during the observation period. Person years for 1985 were estimated as half the number of live births for this year (5,539 × 0.5 = 2,770), person years for 1986 as half the number of live births for this year (5,959 × 0.5) + the number of live births for 1985 (5,539) = 8,519, person years for 1987 as half the number of live births for this year (6,243 × 0.5) + live births for 1985 + 1986 (5,539 + 5,959) = 14,620, and so forth (Figure 1). These numbers are approximations for the at-risk population, as migration of individuals in and out of Oslo was not accounted for.

In order to compare our results with other investigations, we also calculated cumulative incidences per 1000 live births. This was the number of cases divided by the total number of live births in Oslo during 1985 to 2003. The surveillance period for case registration in this study was between 1.1.2004 and 1.8.2006.

### Statistics

The Epi Info 6 statistical software was used for data entry and analysis of frequency distribution of variables. Population statistics for Oslo were provided by the Medical Birth Registry of Norway. The relative risk of PE according to time period and age at onset of disease was estimated with Poisson regression using STATA 9.0. Confidence intervals (CI) for incidence rates were calculated according to Greenland & Rothman [17], while CI for proportions were based on the Poisson distribution [18]. Odds ratios (OR) were calculated using contingency tables, and p-values were calculated using Fisher's exact test. Differences between median values of age were calculated using the Mann-Whitney U test.

### Ethics

The Norwegian Social and Health Directorate and the Regional Ethics Committee approved the study.

## Results

**Figure 2:**
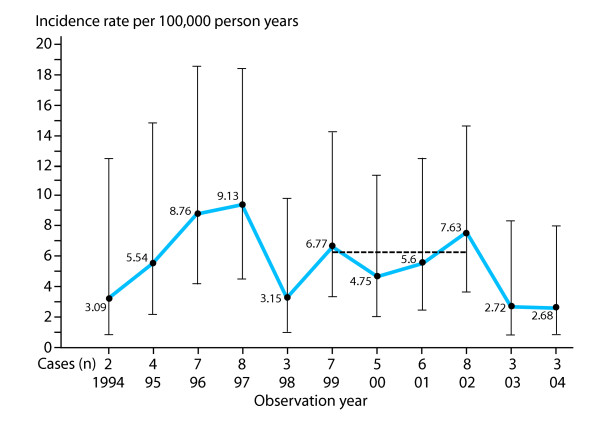
**Incidence rates of progressive encephalopathy**. The incidence rates during the last 10 years of the observation period are shown. Before 1994, the incidence rates were overestimated due to artificially low numbers of person years. The incidence rates for 2003 and 2004 may be underestimated due to short observation time. However, a time trend analysis using incidence rate ratios did not demonstrate any trend changes (data shown in Table 2). During the most representative period, 1999 to 2002, the mean incidence rate was 6.13 per 100,000 person years (horizontal line).

We found 84 PE cases among 1,305,997 person years, giving an overall incidence rate of 6.43 per 100,000 person years (95% CI 5.15–7.97). The mean incidence rate between 1999 and 2002, the most representative part of the observation period, was 6.13 per 100,000 person years (95% CI 4.17–9.0) (Figure 2). There was no significant time trend in incidence, but there was a strong and significant reduction in the risk of PE with increasing age of the child (Table 2). Likewise, when the incidence rates for metabolic and neurodegenerative cases were assessed separately, there were no significant time trend changes (data not shown). The age-specific incidence rates per 100,000 were: 79.89 (<1 year), 8.64 (1–2 years), 1.90 (2–5 years), and 0.65 (>5 years) (95% CI not shown). In a population with equal number of children in each birth cohort (age standardization), this would correspond to an average incidence rate of about 6.4 new cases per 100,000 person years for the childhood population 0 to 15 years of age. The proportions of the various aetiological categories and their overall incidence rates calculated for the whole observation period are shown in Table 3. There were 56 boys (66.7%) (95% CI 55.5–76.6) and 28 girls (33.3%) (95% CI 23.4–44.5).

**Table 2:** Relative risks of progressive encephalopathy according to time period and age estimated as incidence rate ratios with 95% confidence intervals using Poisson regression

	Incidence rate ratios
**Time periode**	

1985–89	1.0 (reference value)
1990–94	0.84 (0.41–1.73)
1995–99	1.39 (0.73–2.65)
2000–04	1.11 (0.57–2.16)

**Age (y)**	

<1	1.0 (reference value)
1–1.99	0.11 (0.06–0.20)
2–5	0.02 (0.01–0.05)
>5	0.01 (0.00 – 0.02)

**Table 3:** Aetiological classification and incidence rates in children with progressive encephalopathy

	n	%	95% CI	IR per 100,000 person years^a^	95% CI
I. Metabolic	55	65.5	54.3–75.5	4.21	3.23–5.49
A. Subcellular organelles	28	33.3	23.4–44.5	2.14	1.48–3.11
Lysosomal	23	27.4	18.2–38.2	1.76	1.17–2.65
Mitochondrial	3	3.6	0.7–10.1	0.23	0.07–0.71
Peroxisomal	2	2.4	0.3–8.3	0.15	0.04–0.61
B. Intermediate metabolism	27	32.1	22.4–43.2	2.07	1.42–3.02
Organic aciduria	11	13.1	6.7–22.2	0.84	0.47–1.52
Fatty acid oxidation defect	6	7.1	2.7–14.9	0.46	0.21–1.02
Urea cycle disorders	4	4.8	1.3–11.7	0.31	0.12–0.82
Galactosemia	4	4.8	1.3–11.7	0.31	0.12–0.82
Unspecified	2	2.4	0.3–8.3	0.15	0.04–0.61
II. Neurodegenerative	27	32.1	22.4–43.2	2.10	1.42–3.02
A. Specified	10	11.9	5.9–20.8	0.77	0.41–1.42
B. Unspecified	17	20.2	12.3–30.4	1.30	0.81–2.09
III. Infectious	2	2.4	0.3–8.3	0.15	0.04–0.61
Total	84	100	-	6.43	5.19–7.97

CI: confidence interval; IR: incidence rates

^a^Not adjusted for time periods

The overall cumulative incidence of PE among 138,550 live births was 0.60 per 1000 (95% CI 0.47–0.73). Other cumulative incidences were: metabolic diseases 0.40 (95% CI 0.30–0.50), lysosomal storage disorders 0.17 (95% CI 0.10–0.24), and organic acidurias combined with fatty acid oxidation defects 0.12 (95% CI 0.06–0.18), per 1000 live births.

The age of onset of disease was distributed as neonatal in 32.1% (27/84), infantile in 39.3% (33/84), early juvenile in 23.8% (20/84), and juvenile in 4.8% (4/84) (Table 4). None of the cases had late juvenile onset. 71.4% (60/84) of the cases presented during the first year of life. In the metabolic group 45.5% (25/55) had neonatal onset, compared to 7.4% (2/27) in the neurodegenerative group (p < 0.001). The median age at the time of diagnosis in the neurodegenerative group was 4.5 years (25^th ^– 75^th ^quartiles 1.8 to 10.0 years), compared to 0.5 years (25^th ^– 75^th ^quartiles 0.02 to 3.0 years) in the metabolic group (p < 0.001).

**Table 4:** Age at the onset of symptoms of disease in children with progressive encephalopathy

	0–4 weeks		1–12 months		1–5 years		6–12 years		Total	
	
	n	%	n	%	n	%	n	%	n	%
Metabolic	25	45.5	12	21.8	15	27.2	3	5.5	55	100
Neurodegenerative	2	7.4	19	70.4	5	18.5	1	3.7	27	100
Infectious^a^	0	0.0	2	100	0	0.0	0	0.0	2	100
Total	27	32.1	33	39.3	20	23.8	4	4.8	84	100

^a^HIV encephalopathy

The 28 different diagnoses assigned to the individual cases (Table 5) included patients with rare and novel diseases, such as congenital neuronal ceroid lipofuscinosis caused by mutations in the Cathepsin D gene [19], 2-methylbutyrylglycinuria [20] confirmed by mutation analysis (Andresen B, personal communication), and Cockayne syndrome caused by a novel mutation in the *CSA *gene [21]. The neurodegenerative group included three patients with congenital microphthalmia and brain atrophy [22] and one patient with a phenotype compatible with pontocerebellar hypoplasia/atrophy and spinal muscular atrophy [23]. Two patients with fatal HIV encephalopathy were included, while cases with vCJD, SSPE, and juvenile Huntington disease were not found. Patients with glycosylation defects were not identified during the observation period. However, two siblings had presented with this type of disorder in 1984 [24].

**Table 5:** Diagnoses in 84 children with progressive encephalopathy

	Diagnoses (n)
Lysosomal	I cell disease (1), alpha-Mannosidosis (1), MLD (3), MPS1 (4), MPS2 (1), MPS3 (1), NCL congenital [19] (3), NCL3 (4), NPC (3), Salla disease (1), Sandhoff disease (1)
Mitochondrial	Leigh disease (3)
Peroxisomal	Adrenoleukodystrophy X-linked (2)
Organic aciduria	2-methylbutyryl CoA dehydrogenase deficiency [20]^a ^(1), glutaric aciduria (1), L2 hydroxy glutaric aciduria (2), methyl malonic aciduria^b ^(2), multiple carboxylase deficiency (2), propionic aciduria (3)
Fatty acid beta oxidation	MTP (2), MCAD (2), VLCAD (1), unspecified (1)
Urea cycle	CPS1 (3), OCT (1)
Other	Galactosemia (4), Unspecified intermediate metabolism (2)
Neurodegenerative, specified	Ataxia teleangiectasia (1), Cockayne syndrome (2), megaloencephalic leukoencephalopathy with subcortical cysts (1), microphthalmia brain atrophy disease [22] (3), pontocerebellar hypoplasia-infantile spinal muscular atrophy [23] (1), Schinzel Gideon syndrome (2)
Neurodegenerative, unspecified	Mainly affecting: basal ganglia (1), cerebellum (8), cerebellum and basal ganglia (1), cerebellum and brain stem (1), cerebral cortex (3), cerebral white matter (3)
Infectious	HIV encephalopathy (2)

CPS: carbamyl phosphate synthetase; HIV: human immune deficiency virus; MCAD: medium-chain acyl CoA dehydrogenase; MLD: metachromatic leukodystrophy; MPS: mucoplysaccharidosis; MTP: mitochondrial trifunctional protein; NCL: neuronal ceroid lipofuscinosis; NPC: Niemann Pick disease type C; OCT: ornithine transcarbamylase; VLCAD: very-long-chain acyl CoA dehydrogenase

^a^The clinical course in this disease is uncertain

^b^Enzymatic diagnosis unknown

## Discussion

Our investigation showed that 6.0 to 6.5 per 100,000 children between 0 and 15 years of age presented with signs of PE annually. From 1999 on the population was complete and included all person years between 0 and 15 years at risk for getting PE. However, the incidence rates from 2003 and 2004 may be underestimated, as the observation time for children born in 2003 was short. Thus, the most representative part of the observation period was between 1999 and 2002 with an average incidence rate of 6.13 per 100,000 person years (see Figure 2). There was a strong tendency for PE to present early in life, as 32.1% of the cases presented before 4 weeks and 39.3% presented between 1 and 12 months of age (see Table 4). This corresponded to an incidence rate of almost 80 per 100,000 person years below 1 year, approximately 13 times more common than the mean incidence rate in the total childhood population. The male to female ratio of 2 could not be explained by known X-linked inherited disorders, which occurred in only four boys in our study. However, males outnumber females with regards to several types of neurological handicaps, such as mental retardation [25] and autism [26].

Incidence rates expressed per person years have not been used as a measure of frequency of PE by other investigators. Consequently, the frequency of known diagnoses in our study was not directly comparable with similarly designed epidemiological studies. However, the cumulative incidence of 0.60 per 1000 live births was close to that of 0.58 per 1000 live births in West Sweden [12]. For metabolic diseases, the cumulative incidence of 0.40 per 1000 (95% CI 0.30–0.50) was somewhat higher than 0.27 per 1000 live births (95% CI 0.269–0.271) in a large-scale Italian study comprising 200 diseases [27]. We did not include phenylketonuria, which has a cumulative incidence of 7.5 per 100,000 live births in Norway [28]. The cumulative incidence of mitochondrial encephalopathy in our study was as low as 2.2 per 100,000 live births, approximately one-third of the cumulative incidence found in Sweden [29]. With a historic cohort design, we were dependent on the information given in the medical charts and may therefore have missed some accuracy regarding diagnoses, particularly for the early years of the observation period. Mitochondrial disorders may have been inadequately diagnosed in Norway. The cumulative incidence of lysosomal storage diseases of 0.17 per 1000 (95% CI 0.10–0.24) in our study was comparable to 0.13 per 1000 in Western Australia [30] and 0.25 per 1000 in Portugal [10], while the combination of organic acidurias and fatty acid oxidation defects of 0.12 per 1000 (95% CI 0.06–0.18) in our study was similar to that of 0.13 per 1000 live births in Germany [31]. There was a trend towards identifying an exact molecular cause in patients with known diseases. The potentially increased incidence rate of PE in the non-western immigration population, associated with higher proportion of consanguineous marriages, will be addressed in a separate study.

The neurodegenerative group comprised 32.1% (95% CI 22.4–43.2%) (see Table 3) of all cases, which is comparable to the proportion (28%) in West Sweden [12]. However, we did not include Rett syndrome in our study. The proportion of cases with unspecified neurodegenerative diagnosis of 20.2% (95% CI 12.3–30.4) occurred at an incidence rate of 1.3 per 100,000 person years. The incidence rate of this heterogeneous group has not previously been reported in the literature. Despite extensive investigation, this group may contain patients with rare metabolic diseases or metabolic deficiencies with an unusual presentation. For example, one patient initially classified as having an unspecified neurodegenerative disorder with cerebellar atrophy was eventually diagnosed with juvenile Sandhoff disease [32] at the age of 15 years. The cerebellum and the basal ganglia are frequently targeted by metabolic deficiency or other genetically determined disorders. The majority of our unspecified neurodegenerative cases demonstrated neural loss in these particular areas.

The clinical course in the neurodegenerative group, both known and unspecified, appeared generally less aggressive than for those in the metabolic group. The proportion with neonatal onset was significantly less compared to the metabolic group. Also, the median age of diagnosis was considerably increasesed in the neurodegenerative compared to the metabolic group. On the other hand, the prospect of therapeutic measures is almost non-existent relative to the increasing number of metabolic diseases which have become amenable to treatment, such as lysosomal storage disorders [33].

## Conclusion

The incidence rate of PE in children was estimated to be between 6.0 and 6.5 per 100,000 person years, while the cumulative incidence was 0.6 per 1000 live births. Almost two-thirds of the cases were metabolic disorders, and one-third other neurodegenerative diseases. Unspecified neurodegenerative diseases accounted for one-fifth of all cases. There was a strong reduction in the risk of PE with increasing age. This trend was most noticeable in the metabolic group, in which less than half had neonatal presentation. The neurodegenerative group was characterized by a later onset of symptoms and a markedly older age at the time of diagnosis.

## Abbreviations

CI: confidence interval

ERD: electronic registry of diagnoses

ICD: international classification of disease

MRI: magnetic resonance imaging

OR: odds ratio

PE: progressive encephalopathy

## Competing interests

The authors declare that they have no competing interests and that all have read and approved the manuscript.

## Authors' contributions

PS planned and designed the study, identified cases, and drafted the manuscript. OJK identified a large number of cases and participated in the design of the study. MA contributed to the creation of the electronic database in the Epi Info 6 statistical software used for data entry and analyses. BW assisted in the identification and classification of the metabolic cases and contributed to drafting the manuscript. TR assisted in the identification and classification of the metabolic cases and in drafting the manuscript. JD contributed to the identification of cases and was responsible for the diagnostic investigations of the neurodegenerative cases at Ullevål University Hospital. BB performed the search for cases in the EDR. FS contributed to the identification of cases and was responsible for diagnostic investigations in neurodegenerative cases at Aker University Hospital. PM contributed to study design, statistical analyses, data interpretation, and drafting of the manuscript. All authors read and approved the final manuscript

## Pre-publication history

The pre-publication history for this paper can be accessed here:


